# Molecular Systematics of the Cape Parrot (*Poicephalus robustus*): Implications for Taxonomy and Conservation

**DOI:** 10.1371/journal.pone.0133376

**Published:** 2015-08-12

**Authors:** Willem G. Coetzer, Colleen T. Downs, Mike R. Perrin, Sandi Willows-Munro

**Affiliations:** School of Life Sciences, University of KwaZulu-Natal, Pietermaritzburg, South Africa; University of British Columbia Okanagan, CANADA

## Abstract

The taxonomic position of the Cape Parrot (*Poicephalus robustus robustus*) has been the focus of much debate. A number of authors suggest that the Cape Parrot should be viewed as a distinct species separate from the other two *P*. *robustus* subspecies (*P*. *r*. *fuscicollis* and *P*. *r*. *suahelicus*). These recommendations were based on morphological, ecological, and behavioural assessments. In this study we investigated the validity of these recommendations using multilocus DNA analyses. We genotyped 138 specimens from five *Poicephalus* species (*P*. *cryptoxanthus*, *P*. *gulielmi*, *P*. *meyeri*, *P*. *robustus*, and *P*. *rueppellii*) using 11 microsatellite loci. Additionally, two mitochondrial (cytochrome oxidase I gene and 16S ribosomal RNA) and one nuclear intron (intron 7 of the β-fibrinogen gene) markers were amplified and sequenced. Bayesian clustering analysis and pairwise F_ST_ analysis of microsatellite data identified *P*. *r*. *robustus* as genetically distinct from the other *P*. *robustus* subspecies. Phylogenetic and molecular clock analyses on sequence data also supported the microsatellite analyses, placing *P*. *r*. *robustus* in a distinct clade separate from the other *P*. *robustus* subspecies. Molecular clock analysis places the most recent common ancestor between *P*. *r*. *robustus* and *P*. *r*. *fuscicollis* / *P*. *r*. *suahelicus* at 2.13 to 2.67 million years ago. Our results all support previous recommendations to elevate the Cape Parrot to species level. This will facilitate better planning and implementation of international and local conservation management strategies for the Cape Parrot.

## Introduction

Accurate species delimitation plays an important role in effective conservation of biodiversity and assisting conservation authorities with the planning and implementation of appropriate conservation strategies. The utility of subspecies in conservation has been a subject of controversy for a long time [1–5]. It has been shown that in some cases subspecies do not form separate phylogenetic clusters and classifying taxa to subspecies rank can be misleading [2]. Subsequently, subspecies are not always given the same conservation consideration as species, especially with less well studied taxa, which can hinder protection of genetically distinct lineages [4, 6]. This is particularly relevant to birds where Phillimore and Owens [1] estimated that roughly 36% of traditionally defined avian subspecies from North America and Eurasia form distinct phylogenetic clusters. It is therefore important to critically identify such phylogenetically distinct lineages and where appropriate elevate subspecies to species so that they can be given adequate conservation consideration.

The genus *Poicephalus* (Psittaciformes) is the most species rich and widely distributed parrot genus in Africa. *Poicephalus* consists of nine species which are divided into two clades [7, 8]. These are the *P*. *robustus* clade, including the Cape Parrot (*P*. *robustus*) and the Red-fronted Parrot (*P*. *gulielmi*); and the *P*. *meyeri* clade, consisting of the Yellow-faced Parrot (*P*. *flavifrons*), the Senegal Parrot (*P*. *senegalus*), the Red-bellied Parrot (*P*. *rufiventrisi)*, the Niam-niam Parrot *(P*. *crassus*), the Brown-headed Parrot (*P*. *cryptoxanthus*), Meyer’s Parrot (*P*. *meyeri*) and Ruppell’s Parrot (*P*. *rueppellii*) [9]. Several of the species (*P*. *robustus*, *P*. *gulielmi*, *P*. *senegalus*, *P*. *flavifrons* and *P*. *meyeri*) are further divided into subspecies. This study will focus on *Poicephalus robustus* which is currently divided into three recognised subspecies, namely the Cape Parrot (*P*. *r*. *robustus*), the Grey-headed Parrot (*P*. *r*. *suahelicus*) and the Brown-necked Parrot (*P*. *r*. *fuscicollis*).

**Fig 1:**
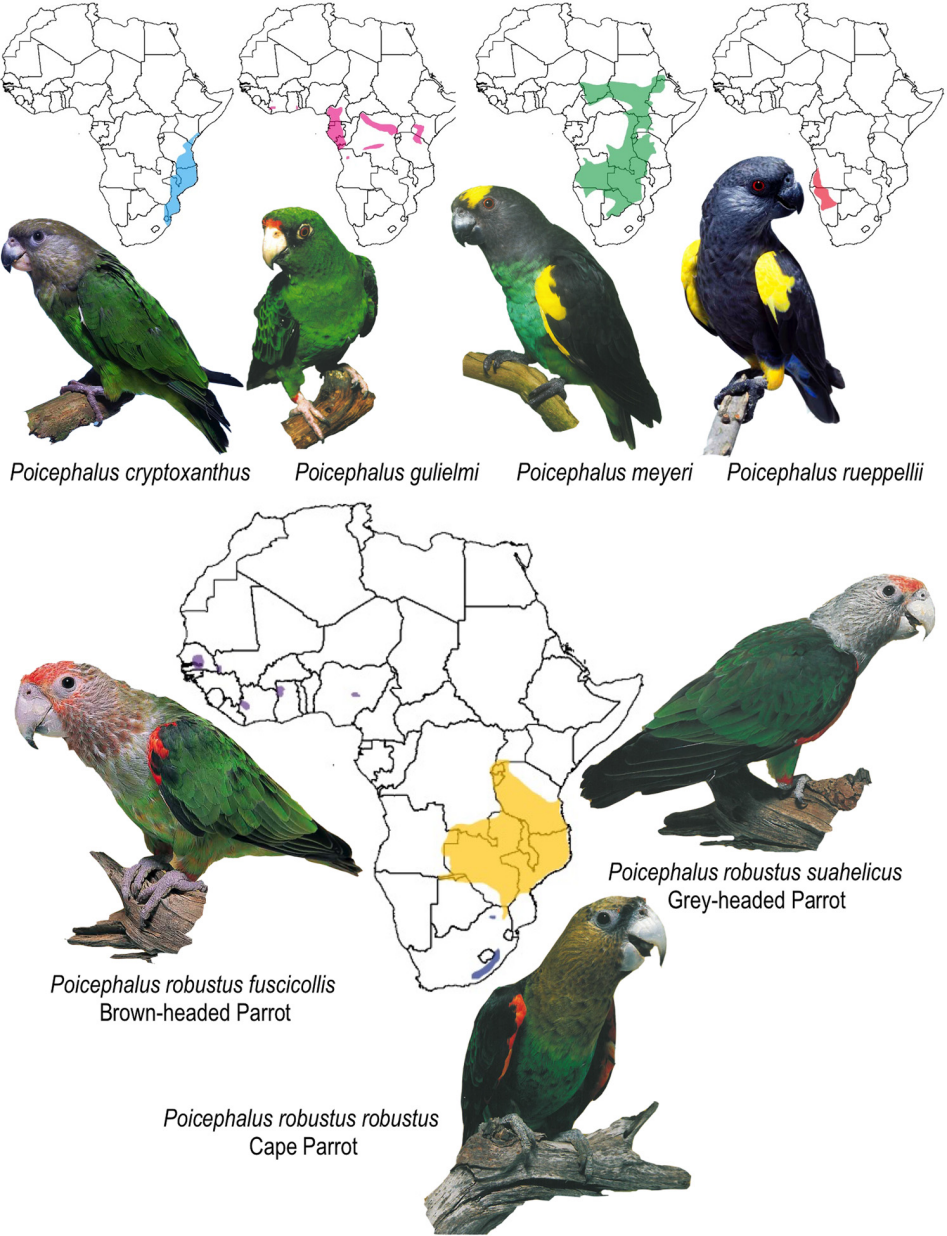
Distribution map of the *Poicephalus* species (and *P*. *robustus* subspecies) included in the study (maps redrawn from Perrin 2012, photos used with permission from Cyril Laubscher).

The recognition of the South African taxon *P*. *r*. *robustus* as a species separate from *P*. *r*. *fuscicollis* and *P*. *r*. *suahelicus* has been a controversial subject over the last few decades [10–13]. The *P*. *robustus* clade exhibits an allopatric geographical distribution, with most species restricted to forest habitats [9]. Within the *P*. *robustus* group, the west African *P*. *r*. *fuscicollis* is found in Red Mangrove forests, mature wooded savannah and palm woodlands [10, 12]. This species was once widely distributed from Angola through to West Africa but is now primarily found in Gambia [13]. The subspecies *P*. *r*. *suahelicus* occurs in a wide variety of woodland habitats and is widely distributed in the south-eastern region of the Democratic Republic of Congo, south-western Uganda, Rwanda, north-western Tanzania, Malawi, Zambia, Mozambique, Zimbabwe and the northern Limpopo province of South Africa [7, 14, 15]. The South African subspecies *P*. *r*. *robustus*, however, is a habitat specialist and is almost exclusively restricted to the southern mistbelt (*Afrocarpus*/*Podocarpus*) forests of southern Africa (Fig 1) [12]. The distribution of the subspecies *P*. *r*. *robustus* and *P*. *r*. *suahelicus* are reported to overlap in the Limpopo Province of South Africa, but there is strong evidence that the two taxa are ecologically separated by habitat and altitude with *P*. *r*. *robustus* found in mixed *Afrocarpus/Podocarpus* mistbelt forests above 1000 m and *P*. *r*. *suahelicus* preferring mixed woodland habitats below 800 m [11].

Morphologically *P*. *r*. *robustus* differs significantly from the other two *P*. *robustus* subspecies. In addition to differences in plumage colouration [10, 13, 14], *P*. *r*. *robustus* is the smallest of the three subspecies and has a more lightly structured bill than either *P*. *r*. *fuscicollis* or *P*. *r*. *suahelicus* (Fig 1). Wirminghaus et al. [13] showed that there are significant differences in skull dimensions between *P*. *r*. *robustus* and *P*. *r*. *suahelicus*. The two taxa also have quite distinct behaviour and show differences in vocalisations [16, 17], feeding and breeding behaviour [18, 19]. *P*. *r*. *robustus* is a dietary specialist, feeding predominantly on *Afrocarpus/Podocarpus* fruits, they also prefer these trees for nesting during the breeding season (August to February) [18, 20]. *P*. *r*. *suahelicus*, however, is a dietary generalist feeding on a range of forest fruits and seeds, they also prefer to nest in *Adansonia* trees, and breed from April to August [19, 21].

Given the morphological, ecological and behavioural differentiation of the three *Poicephalus* subspecies, several authors have proposed full species status for the South African *P*. *r*. *robustus* [10, 11, 13]. However, international conservation organizations, such as BirdLife International, have highlighted the need for genetic data for full species status to be recognized. In particular genetic data is needed to clarify if overlapping populations of *P*. *r*. *robustus* and *P*. *r*. *suahelicus* are reproductively isolated.

The conservation of subspecies is a contentious issue, especially given the limited resources currently available for biodiversity conservation. The International Union for Conservation of Nature (IUCN) considers the taxonomic rank of species as the primary unit for conservation [22] and rarely assesses the status of subspecies [4]. This is problematic for *P*. *r*. *robustus*, given that the population has undergone a dramatic decline in the last century with an estimated current population size of less than 1600 birds in the wild [23]. Up to five decades ago Cape Parrots were recorded in many of the forests of KwaZulu-Natal, but these birds are now only rarely sighted [24]. This population decline has been attributed to various factors, including habitat loss, illegal harvesting of wild birds and psittacine beak and feather disease [17, 23, 24]. Based on previously published morphological, behavioural and ecological data [10, 11, 13], *P*. *r*. *robustus* is considered a distinct conservation unit by the International Ornithologists’ Union and BirdLife South Africa and is accordingly listed for protection under South African legislation [25]. It is also listed as Endangered in the Red Data Book of birds of South Africa, Lesotho and Swaziland [26]. The Cape Parrot is, however, only listed as Least Concern in the IUCN Red List of Threatened Species [27] as the Red List assessment does not recognize *P*. *r*. *robustus* as a species separate from the more widespread Grey-headed Parrot (*P*. *r*. *suahelicus*) and the Brown-necked Parrot (*P*. *r*. *fuscicollis*) [12]. In accordance with the IUCN Red List and the position taken by BirdLife International, international trade of *P*. *r*. *robustus* under CITES (Convention on International Trade in Endangered Species of Wild Fauna and Flora) is regulated at the species level, whereby no distinction is made between the three *P*. *robustus* subspecies, effectively ignoring the poor conservation status of this South African endemic. Effective regulation and monitoring of the international trade in *P*. *r*. *robustus* is therefore impossible. To ensure effective conservation of *P*. *r*. *robustus*, both locally and internationally, it is important to provide convincing scientific evidence that *P*. *r*. *robustus* warrants species status. Our main aim was to assess the taxonomic status of *P*. *r*. *robustus* using both multilocus microsatellites and mitochondrial data. We predicted that *P*. *r*. *robustus* would be genetically distinct from *P*. *r*. *fuscicollis* and *P*. *r*. *suahelicus*, thus supporting elevation to full species status that has already been accepted by some authorities on the basis of morphological, ecological and behavioural data [25, 26]. The use of multilocus analyses is a well established method of separating closely related bird species [28–31] and investigating within species relationships [22, 32–34].

## Materials and Methods

### Ethics

We obtained ethical approval for this study from the University of KwaZulu-Natal Animal Ethics sub-committee (Ref numbers: 074/13/Animal, 017/14/Animal). The sampling permits for KwaZulu-Natal were obtained through Ezemvelo KZN Wildlife (Permit number: OP 1546/2014). All necessary import permits were obtained through Ezemvelo KZN Wildlife (Permit numbers: OP 1230/2014 and OP 878/2014) or the National Department of Environmental Affairs (Permit Number: 133120).

### Sampling

We included a total of 138 *Poicephalus* specimens representing the four southern African species (*P*. *cryptoxanthus*, *P*. *meyeri*, *P*. *robustus* and *P*. *rueppellii*) and one central African species (*P*. *gulielmi*; S1 Table) in our analyses. These species were selected based on their distribution ranges either overlapping or being in close proximity to *P robustus ssp*. We included representatives of all three *P*. *robustus* subspecies (*P*. *r*. *fuscicollis*, *P*. *r*. *robustus* and *P*. *r*. *suahelicus*) and two of the *P*. *gulielmi* subspecies (*P*. *g*. *gulielmi* and *P*. *g*. *massaicus*). We had multiple representatives of *P*. *r*. *robustus* (n = 32) drawn from all three of the isolated South African populations (Eastern Cape = 10, KwaZulu-Natal = 13 and Limpopo = 9). We did this to ensure the inclusion of as much genetic variation across the *P*. *r*. *robustus* distribution range as possible.

We used a variety of different tissue types. Whole blood collected from wild trapped and captive bred birds was stored on Whatman FTA Elute or Classic Cards. Clean needles were used for each individual to avoid cross-contamination of blood samples. Feathers were collected from the field, and muscle tissue samples were taken from dead birds. Archival museum toe pad samples were sourced from various local and international museums (S1 Table).

### DNA extraction

For the whole blood stored on Whatman FTA Elute cards, we followed the standard DNA extraction protocol as suggested by the manufacturer. The DNA was eluted with 30 *μ*l ultrapure water and stored at -20°C. We extracted DNA from the muscle tissue samples using the NucleoSpin Tissue kit (Macherey-Nagel), following the manufacturers standard protocol. Modified extraction protocols were used for the toe pad and feather samples. In order to minimize surface contamination, we performed three washing steps (with 95% ethanol, 70% ethanol and ultrapure water) prior to extraction, followed by a final hydration step where samples were soaked in 1 ml ultrapure water for 60 min. Thereafter we extracted DNA using the NucleoSpin Tissue kit. The lysis step was extended until the samples were completely lysed. The final elution step was also modified, such that after 40 μl of preheated elution buffer was added to the spin column the samples were incubated at 70°C for 10 min. After centrifuging the samples at 11 000 x g the elution buffer was placed back into the same spin column and an additional 40 μl warmed elution buffer added to each tube. We incubated the samples at 70°C for 5 min, after which we centrifuged them at 11 000 x g to obtain the final DNA product.

### Microsatellite amplification

We chose a panel of 11 microsatellite loci (*Prob06*, *Prob15*, *Prob18*, *Prob23*, *Prob25*, *Prob26*, *Prob28*, *Prob29*, *Prob30*, *Prob34* and *Prob35*), previously described by Pillay et al. [35], for amplification by polymerase chain reaction (PCR). In each case the forward primers were synthesized with a fluorescent dye on the 5’ end. We divided the microsatellite panel into four multiplex sets (Multiplex 1: *Prob06*, *Prob15* and *Prob26*; Multiplex 2: *Prob29*, *Prob34* and *Prob35*; Multiplex3: *Prob18* and *Prob25*; Multplex4: *Prob23* and *Prob28*), with the locus *Prob30* in a single reaction. The PCR reactions for the fresh samples (blood and muscle tissue samples) consisted of: ~2–30 ng template DNA, 5 μl KAPA2G Fast Multiplex mix (KAPA Biosystems), 0.2 μM of each primer and dH_2_O to give a final reaction volume of 10 *μ*l. The PCR reaction mixtures for the feather and archival toe pad samples consisted of: ~20–200 ng template DNA, 5 μl KAPA2G Fast Multiplex mix, 0.2 μM of each primer, 0.3 μl of 1 mg/ml BSA and dH_2_O to give a final reaction volume of 10 *μ*l. We used identical PCR cycle parameters for all multiplex reactions and included an initial denaturation step at 94°C for 3 min followed by 30 cycles at 94°C for 30 s, 60°C for 30 s, 72°C for 30 s, with a final extension step at 72°C for 5 min. The PCR cycles were increased to 40 cycles for the museum and feather samples to ensure sufficient amplification. PCR setup prior to addition of the DNA was done in a DNA free area to avoid contamination of reagents.

We sent all amplified products to the Central Analytical Facility at Stellenbosch University, South Africa for fragment analysis. The software program Gene Marker v2.4.0 (Soft Genetics) was used for subsequent genotype scoring. To ensure genotyping consistency, we reamplified the archival museum samples and analysed each locus three times. In addition we reamplified 20% of the fresh samples to check for consistency in genotype scoring.

### DNA sequencing

In addition to the microsatellite analysis, we amplified and sequenced two mitochondrial (mtDNA) markers and one nuclear intron (nucDNA) marker: cytochrome oxidase I (COI using the primers BirdF1/BirdR1; [36]), 16S ribosomal RNA (16S rRNA using the primers 16Sa/16SB; [37]) and a nuclear intron of the β-fibrinogen gene (β-fib using the primers FIB-BI7U/FIB-BI7L; [38]). Where possible, these three markers were amplified for five representative *P*. *r*. *robustus* samples (Eastern Cape, KwaZulu-Natal and Limpopo) and two samples for each of the other species and subspecies included in the microsatellite analysis (See S1 Table). PCR reactions for COI and 16S rRNA consisted of: ~20–150 ng template DNA, 2.5 μl 10 x KAPA buffer, 1 U KAPA Taq DNA polymerase, 200 μM dNTPs, 0.2 μM of each primer and 18.4 μl dH_2_O to give a final reaction volume of 25 *μ*l. We added an additional 0.5 mM MgCl_2_ to the reaction mixture for β-fib. A touchdown PCR protocol was used. The PCR cycle parameters for COI and 16S rRNA included an initial denaturation step at 95°C for 3 min followed by 10 cycles at 95°C for 30 s, 60–50°C for 30 s, 72°C for 30 s, 25 cycles at 95°C for 30 s, 50°C for 30 s, 72°C for 30 s with a final extension step at 72°C for 5 min. The touchdown temperature range and annealing temperature for β-fib was 65–55°C and 55°C.

We sent all PCR products which showed positive amplification for sequencing. Cycle sequencing was performed using the BigDye Chemistry, v3.1 and sequencing products were analyzed on an Applied Biosystems 3730xl Genetic Analyzer (Applied Biosystematics, Perkin Elmer). All heterozygous sites in the nuclear intron were coded using the International Union of Biochemistry (IUB) codes. All raw sequence data were viewed and edited in BioEdit v7.1.11 [39]. The edited sequences were aligned using ClustalW [40] as implemented in MEGA v6 [41] and then checked manually to ensure homology. We deposited all new sequences in GenBank (S2 Table). *Psittacus erithacus*, the Grey Parrot, was included as an outgroup with sequences downloaded from GenBank (S2 Table).

### Data analysis

#### Microsatellite analysis

We estimated null allele frequencies for each marker using the software program FreeNA [42] using the Expectation Maximization algorithm (EM) [43]. We compared the null allele corrected and uncorrected global F_ST_ values using the excluding null alleles (ENA) method [42]. Summary statistics (average number of alleles, observed and expected heterozygosity and the number of private alleles), polymorphic information content (PIC), pairwise F_ST_ and analysis of molecular variance (AMOVA) were estimated using GenAlEx v6.5 [44] and Cervus v3.0.7 [45]. Two AMOVA analyses were conducted. One grouping individuals into five species (*P*. *cryptoxanthus*, *P*. *gulielmi*, *P*. *meyeri*, *P*. *robustus* and *P*. *rueppellii)*. The three *P*. *robustus* subspecies and the two *P*. *gulielmi* subspecies were placed into *P*. *robustus* and *P*. *gulielmi* respectively. A second AMOVA was conducted in which the subspecies (*P*. *g*. *gulielmi*, *P*. *g*. *massaicus*, *P*. *r*. *fuscicollis*, *P*. *r*. *robustus* and *P*. *r*. *suahelicus)* were placed into individual groups. We used the program XLSTAT 2014 [46] to generate a 3D principal coordinate analysis (PCoA) figure using pairwise F_ST_ values. Arlequin v3.5 [47] was used to test for linkage disequilibrium and deviation from Hardy–Weinberg equilibrium. We performed Bayesian clustering analysis in STRUCTURE v2.3.4 [48]. Ten independent runs were performed. Each STRUCTURE run consisted of 1,000,000 Markov chain Monte Carlo (MCMC) replicates with a burn-in of 100,000 with the proposed number of clusters (K) ranging from 2 to 10. The no admixture model with correlated allele frequencies was selected for all runs. Sampling locality information was incorporated using the LOCPRIOR model. We used the program STRUCTURE harvester [49] to estimate the most probable number of genetic clusters using the method implemented by Evanno et al. [50]. The STRUCTURE figure and the membership probabilities (Q-values) for each individual and for each cluster were estimated using ClumpAK (http://clumpak.tau.ac.il).

#### DNA sequence analysis

We initially analyzed the three gene regions (COI, 16S rRNA and β-fib) separately and then combined them into a single data matrix. In addition, we analyzed the sequence data from each gene according to origin of marker (mtDNA or nucDNA). Number of variable sites, number of observed transitions, number of observed transvertions and number of observed indels were estimated using MEGA and Arlequin. Phylogenies were constructed using both maximum likelihood (ML) conducted in Garli v2 [51]; and Bayesian inference (BI) using MrBayes v3.2 [52]. For these analyses the optimal model of nucleotide substitution for each gene region was used. This was selected using the Akaike information criterion (AIC) [53] in jModelTest v.2.1 [54]. In the combined analyses, data were partitioned by gene with model parameters unlinked across partitions. In ML analyses branch support was assessed using 1000 bootstraps replicates with consensus topologies generated using PHYLIP v3.695 [55, 56]. Each Bayesian run consisted of three heated chains at default temperature of 0.2 and one cold chain and was run for 10 million generations with the sampling frequency of 1000 and a burn-in of 0.25 (25,000 trees). To ensure that MCMC chains had reached convergence, Tracer v1.5 [57] was used to verify that the appropriate estimated sample sizes (ESS) for all parameters were above 200 [58]. A 50% majority rule consensus tree was constructed in PHYLIP after burn-in was removed. We viewed trees in FigTree v1.4.0 [59]. We calculated pairwise genetic distances in RAxMLGUI v.1.3.1 [60] using the general time reversible nucleotide substitution model with gamma distribution and invariant sites (GTRGAMMAI).

#### Molecular clock analysis

There are no fossil calibration points available within the genus *Poicephalus*. Molecular clock analysis was performed using secondary calibration dates from two other studies [61, 62] to estimate divergence times of *Poicephalus* species. Schweizer et al. [61], using three nuclear genes, used three avian fossil records outside Psittaciformes as calibration points to estimate diversification times. These authors estimated that the separation of Strigopidae from the rest of the parrot taxa occurred ~58.6 million years ago (Mya). White et al. [62] used full mitochondrial genomes and six avian fossil records as calibration points to study the evolutionary history of the Cacatuidae and estimated Strigopidae and the other parrot taxa split ~47.4 Mya.

Given that the divergence dates estimated by Schweizer et al. [61] and White et al. [62] are quite different, we used the calibration points from these two studies in separate analyses. Five calibration points were used from Schweizer et al. [61] and included the split between *Nestor* and the rest of the parrot taxa (58.59 Mya; SD: 8.2), the split between the Australasian Cacatuidae and Psittacidae (47.38 Mya; SD: 7), the split between *Psittacus-Poicephalus* and Arini (35.16 Mya; SD: 5.6), the split between *Amazona*/*Pionus* and *Ara*/*Deroptyus* (25.26 Mya; SD: 5) and the split between *Psittacus* and *Poicephalus* (12.92 Mya; SD: 3.5). We used three calibration points from White et al. [62] and included the split between *Nestor* and the rest of the parrot taxa (47.4 Mya; SD: 7), the split between the Australasian Cacatuidae and Psittacidae (40.7; SD: 7) and the split between *Cacatuninae* and *Calyptorhynchinae* (27.9 Mya; SD: 6).

We downloaded sequences from 27 parrot species covering 21 genera, used by Schweizer et al. [61], from GenBank (S2 Table) and included them in the molecular clock analyses. Divergence times were calculated using BEAST v1.8 [63]. We conducted analyses on two datasets, one containing sequences from all three gene regions (COI, 16S rRNA and β-fib) and then to limit the inclusion of missing data we also analyzed a dataset containing only the mtDNA genes. In all analyses we partitioned the data by gene with the parameters of the substitution models unlinked. The GTR + Γ + I substitution model was used for COI and the GTR + I model was used for β-fib as the best-fit models suggested by jModelTest (TPM2uf + Γ + I and TPM1uf + I) are not currently implemented in BEAST. The GTR + Γ + I model was, however, identified as the best fit model for 16S rRNA. A lognormal relaxed-clock approach was implemented following Schweizer et al. [61] with a Yule speciation model set as tree prior.

For each dataset (three gene and mtDNA only), we conducted two independent simulations for each set of calibration points. Each BEAST run consisted of 400 million generations, with a sampling frequency of 10000 trees. The program Tracer was used to confirm that MCMC chains had reached stationarity and ESS of all parameters were greater than 200. We used TreeAnnotator v1.8.1 (in Beast v1.8.1) to estimate the maximum clade probability tree which we viewed in TreeGraph v2 [64].

## Results

### Microsatellite analysis

In this study we genotyped 138 individuals using 11 microsatellite loci. Individuals in this data set had minimal missing data, with only 3.03% missing data included. Mean null allele frequencies ranged from 0.4% -14.6% across species (Na; S3 Table). In particular the error rate in the data collected from four loci in the *P*. *rueppellii* dataset (*Prob18*, Na = 23.3%; *Prob26*, Na = 22.9%; *Prob29*, Na = 30.2%; *Prob34*, Na = 26.0%) was high, although below values reported in other studies [65]. No loci showed null allele frequencies higher than 30.2%. The detection of null alleles can be biased in natural populations which deviate from Hardy-Weinberg Equilibrium (HWE), as is the case in this study where all loci except *Prob35* deviated from Hardy-Weinberg equilibrium in at least one species/subspecies. The presence of null alleles can inflate F_ST_ values [66], but we found that there was little difference between the global F_ST_ values using the ENA corrected (F_ST_ = 0.25) and the uncorrected (F_ST_ = 0.26) data. The effects of any null alleles present in the *Poicephalus* data set are likely minimal and we performed all further analysis using data from all loci.

Estimates of the mean number of alleles, private alleles, observed and expected heterozygosity are reported in Table 1. All loci were polymorphic in all species/subspecies with the exception of *Prob18* and *Prob30* which were monomorphic in *P*. *g*. *gulielmi* and *Prob25* and *Prob35* which were monomorphic in *P*. *g*. *massaicus* (S3 Table). Ascertainment bias could influence genetic diversity analyses, as allele numbers might be higher in the focal species from which the markers were developed [67]. Noticeably lower allele numbers were only observed in two species (*P*. *rueppellii* and *P*. *gulielmi*). One locus in *P*. *rueppellii* (*Prob6*), two loci in *P*. *g*. *massaicus* (*Prob30* and *Prob25*), and four loci in *P*. *g*. *gulielmi* (*Prob6*, *Prob15*, *Prob18* and *Prob30*) showed allele numbers < 50% of that observed in *P*. *robustus ssp*. Low sample number is also a consideration in the case of *P*. *g*. *gulielmi* (n = 4; S3 Table). Nine of the eleven loci used were highly informative with PIC values > 0.7. The PIC values for each locus range from 0.513 (*Prob35*) to 0.895 (*Prob23*) with a mean PIC value of 0.794. (S3 Table). The highest mean number of alleles was recorded for *P*. *r*. *suahelicus* (N_A_ = 7.091). Private alleles (P_A_) were identified for all species and subspecies. The most distinct species *P*. *rueppellii* has nine private alleles. The number of private alleles observed in the *P*. *robustus* subspecies ranged from one to six alleles, with *P*. *r*. *suahelicus* possessing the highest number of private alleles (P_A_ = 6). The observed heterozygosity (H_O_) ranged from 0.368 to 0.632, and the expected heterozygosity (H_E_) range from 0.457 to 0.705 over all species and subspecies. Of the three *P*. *robustus* subspecies, *P*. *r*. *suahelicus* showed the highest level of genetic diversity (H_E =_ 0.701; Table 1) and is comparable to previous observations (H_E_ = 0.76) [68]. *P*. *r*. *fuscicollis* has a relatively small and fragmented distribution range, and comparatively has the lowest level of genetic diversity among the *P*. *robustus* subspecies (H_E_ = 0.557). This lower genetic diversity estimate might be an artefact of sample size or that the majority of these samples were from captive bred birds, given that Taylor [68] observed a higher level of genetic diversity for *P*. *r*. *fuscicollis* (H_E_ = 0.77). The observed heterozygosity of *P*. *r*. *robustus* (H_O_ = 0.622) was comparable to previous estimates (H_O_ = 0.63) [35].

**Table 1:** Sample details and genetic diversity for each *Poicephalus* species and subspecies analysed. Number of individuals sampled, observed heterozygosity, expected heterozygosity, mean number of alleles and number of private alleles are provided.

Species:	Number of samples:	Average number of alleles:	Observed Heterozygosity:	Expected Heterozygosity:	Number of private alleles:	Polymorphic information content:
*P*. *robustus robustus*	32	5.455	0.622	0.619	1	0.567
*P*. *r*. *suahelicus*	23	7.091	0.632	0.701	6	0.673
*P*. *r*. *fuscicollis*	26	5	0.485	0.557	1	0.519
*P*. *meyeri*	12	5.727	0.515	0.705	6	0.664
*P*. *rueppellii*	16	4.818	0.368	0.581	9	0.536
*P*. *cryptoxanthus*	14	5.909	0.504	0.612	3	0.582
*P*. *gulielmi gulielmi*	4	2.818	0.545	0.457	4	0.412
*P*. *g*. *massaicus*	11	4.364	0.471	0.498	4	0.472
**Total:**	**138**	**5.148**	**0.518**	**0.591**	**-**	**0.794**

### Species delimitation

#### Microsatellite analysis

**Fig 2:**
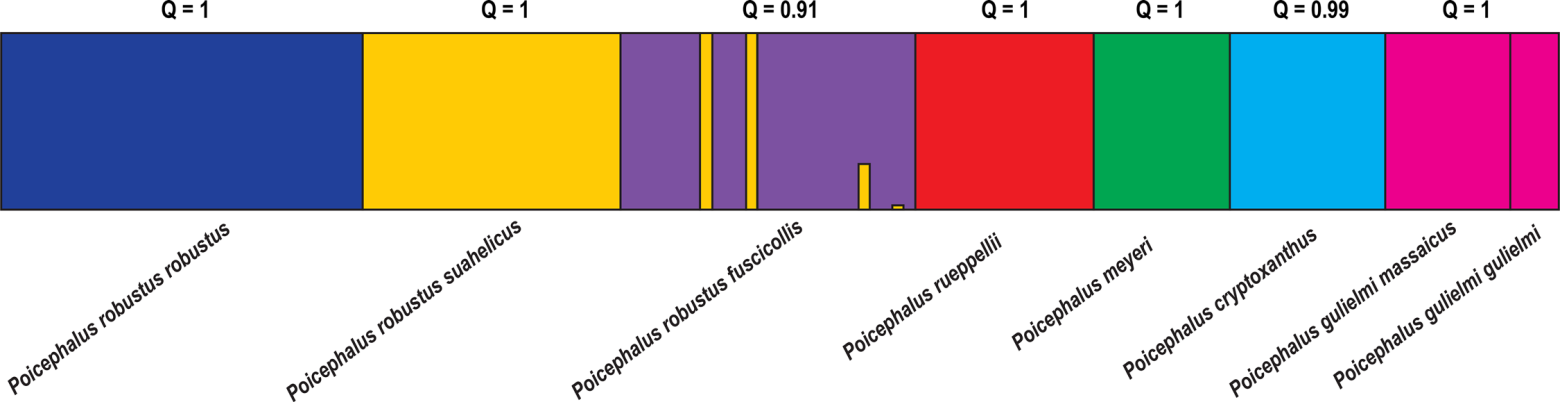
The estimated population genetic structure of the *Poicephalus* species/subspecies used in the current study (K = 7). Each individual is represented by a vertical line, and coloured according to each individual’s estimated membership probability (Q-values). Average Q-values for each cluster is depicted above the figure.

The Bayesian clustering analysis identified seven genetic clusters (K = 7, mean LnP(K) = -4222.4; Fig 2) as the most likely number of clusters following Evanno et al. [50]. These clusters corresponds to the species *P*. *cryptoxanthus*, *P*. *gulielmi* (with *P*. *g*. *gulielmi* and *P*. *g*. *massaicus* clustering together; Q = 1), *P*. *meyeri* and *P*. *rueppelli*. The three *P*. *robustus* subspecies were assigned to separate clusters with only two *P*. *r*. *fuscicollis* individuals assigned to the *P*. *r*. *suahelicus* cluster with high probability (Q = 0.99).

**Fig 3:**
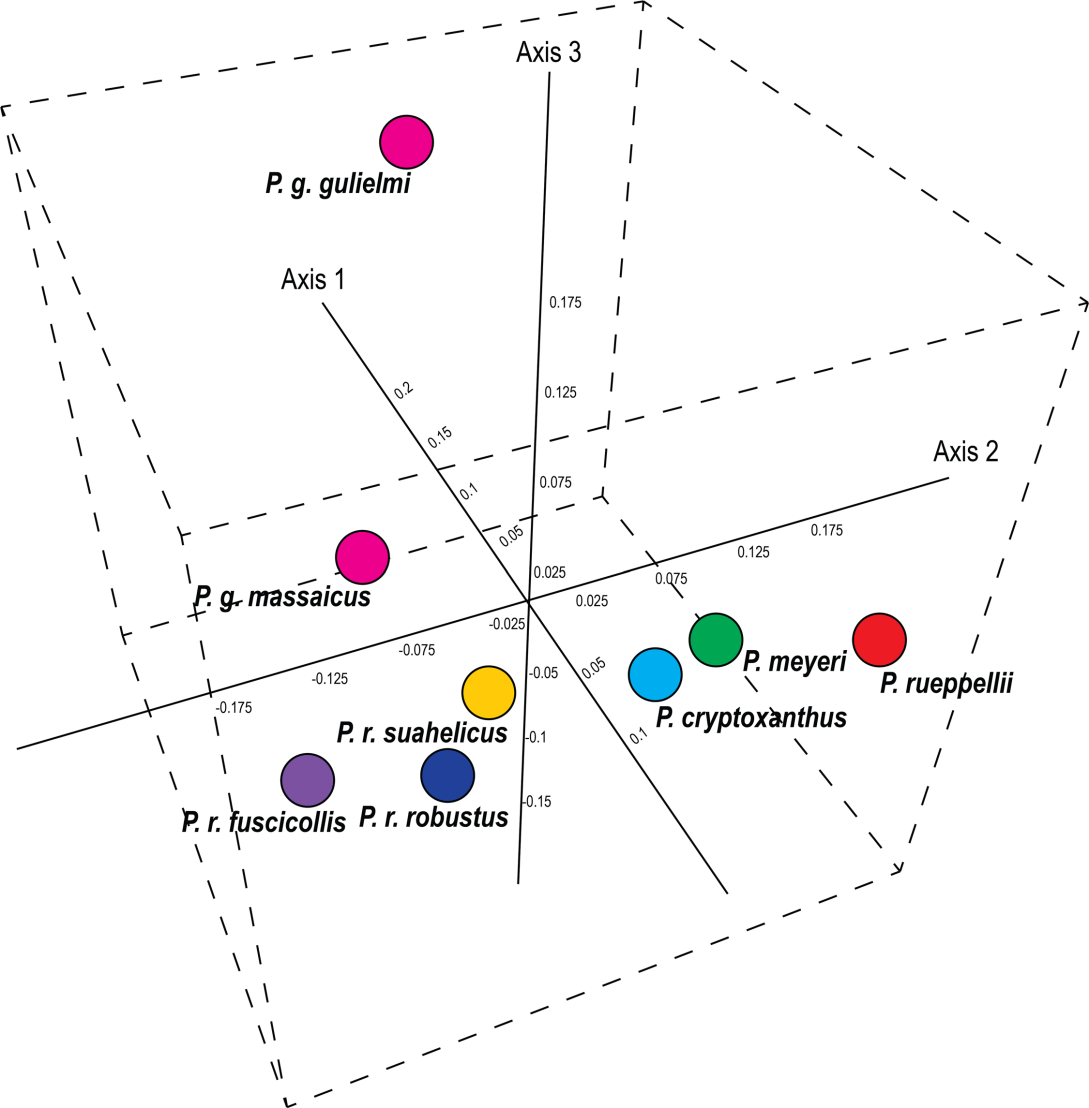
A 3D principal coordinate analysis (PCoA), generated in XLSTAT 2014. The pairwise F_ST_ values estimated between species/subspecies of the *Poicephalus* species included in the study were used to generate the figure. The first three axes explained 70.3% of the estimated variation.

The STRUCTURE clustering was supported by pairwise F_ST_ values which were highly significant between all species and subspecies (0.13 ≤ F_ST_ ≤ 0.41; *P*-value < 0.05). The pairwise F_ST_ values between *P*. *r*. *robustus* and *P*. *r*. *suahelicus* (F_ST_ = 0.14; *P*-value = 0.001), and *P*. *r*. *robustus* and *P*. *r*. *fuscicollis* (F_ST_ = 0.22: *P*-value = 0.001) were comparable to the pairwise values between the other *Poicephalus* species, for example between *P*. *cryptoxanthus* and *P*. *meyeri* (F_ST_ = 0.14; *P*-value = 0.001) and *P*. *cryptoxanthus* and *P*. *rueppelli* (F_ST_ = 0.21; *P*-value = 0.001; S4 Table). These relationships can also be clearly seen in the 3D PCoA drawn from the pairwise F_ST_ values (Fig 3). The global F_ST_ value (subspecies assigned to species) was significantly different from zero (F_ST_ = 0.21; *P*-value = 0.001) and the analysis of molecular variance (AMOVA) indicated that 21% of the observed genetic variation occurred between species with 58% occurring within individuals and 21% among individuals that belong to the same species/subspecies. High F_ST_ values were also recovered when individuals were assigned to subspecies (F_ST_ = 0.25; *P*-value = 0.001). The AMOVA analysis indicated that 25% of the variation occurs between species and subspecies, with 14% variation among individuals. The majority of the genetic variation occurred within individuals (61%).

#### Phylogenetic analysis

The two mtDNA markers were successfully amplified for all 18 specimens; unfortunately β-fib was only successfully sequenced from 10 specimens (S5 Table). The data matrices for each marker included (S5 Table): COI (592 bp; 66 variable sites), 16S rRNA (707 bp; 32 variable sites) and β-fib (707 bp; 4 variable sites). To reduce the effects of missing data we conducted two analyses. Firstly, the data from all three markers including missing data were concatenated and analysed. Secondly, only data from the two mtDNA markers were analysed (no missing data included). There was no significant conflict among the topologies produced when each marker was analysed independently and the data were concatenated (concatenated: 1834 bp, 102 variable characters; mtDNA only: 1127 bp, 98 variable characters). The *P*. *robustus* clade formed a distinct monophyletic group separate from the *P*. *meyeri* clade in both the concatenated (ML bootstrap, 87; Bayes’ posterior probability, 1.00) and mtDNA topologies (ML bootstrap, 94; Bayes’ posterior probability, 1.00), supporting hypotheses proposed by Forshaw [8] and Fry et al. [7].

**Fig 4:**
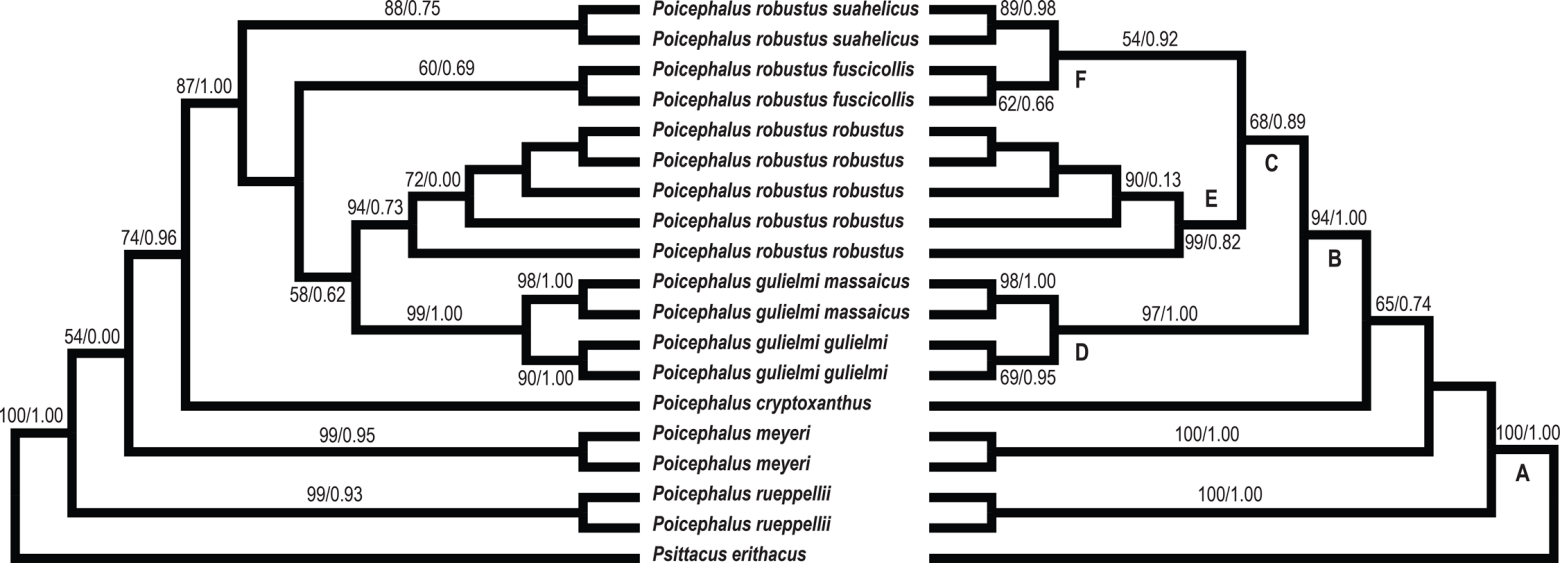
Maximum likelihood phylogeny retrieved of the *Poicephalus* specimens from the current study. The analyses were performed using (left) concatenated data (COI, 16S rRNA and β-fib) and (right) mitochondrial DNA data. Values given above the branches represent maximum likelihood bootstrap values and Bayesian posterior probabilities (in that order). Dated nodes are indicated by numbers next to each node in b) and correspond to Table 2. Species/subspecies were highlighted according to the microsatellite clustering analyses. *Psittacus erithacus* was used as an outgroup.

The phylogenetic analysis confirmed the monophyly of all species with the exception of *P*. *robustus* (Fig 4). Phylogenetic analysis of the mtDNA markers cluster together the subspecies *P r*. *fuscicollis* and *P*. *r*. *suahelicus* (ML bootstrap, 54; Bayes’ posterior probability, 0.92). The phylogenetic position of *P*. *r*. *robustus* is not well resolved. In the concatenated analysis *P*. *r*. *robustus* is placed sister to a clade containing the two *P*. *gulielmi* subspecies although this association is only weakly supported (ML bootstrap, 58; Bayes’ posterior probability, 0.62). In the mtDNA phylogeny the three *P*. *robustus* subspecies are clustered together (ML bootstrap, 68; Bayes’ posterior probability, 0.89).

**Table 2:** Divergence dates of the seven *Poicephalus* species, with *Psittacus erithacus* as outgroup, analysed with a Bayesian lognormal relaxed-clock model. The mean estimated values and the 95% highest posterior density (HPD) ranges are given for the two dataset partitions. The node numbers correspond to Fig 4B. See S1 Fig for the corresponding maximum clade probability trees.

	Divergence times using mtDNA and nuclear gene regions following Schweizer et al. [57]:		Divergence times using mtDNA gene regions only following Schweizer et al. [57]:		Divergence times using mtDNA and nuclear gene regions following White et al. [58]:		Divergence times using mtDNA gene regions only following [58]:	
Node number/ID:	Mean:	95% HPD (Mya):	Mean:	95% HPD (Mya):	Mean:	95% HPD (Mya):	Mean:	95% HPD (Mya):
a / *Poicephalus*	10.4	7.32–13.87	10.34	7.11–14.03	10.27	6.55–15.04	10.63	6.58–15.73
b / *P*. *robustus* clade	6.36	4.12–9.14	6.64	4.14–9.61	6.16	3.68–9.53	6.72	3.88–10.33
c / *P*. *robustus ssp*.	2.24	1.15–3.60	2.67	1.35–4.37	2.13	1.03–3.15	2.62	1.27–4.45
d / *P*. *gulielmi ssp*.	1.9	0.90–3.26	2.01	0.91–3.50	1.81	0.80–3.21	1.97	0.86–3.53
e / *P*. *robustus* population	1.23	0.52–2.16	1.44	0.60–2.63	1.16	0.48–2.11	1.4	0.55–2.61
f / *P*. *r*. *suahelicus-fuscicollis*	0.6	0.16–1.25	0.69	0.17–1.46	0.57	0.14–1.20	0.66	0.16–1.44

The COI sequence differentiation among the *P*. *robustus* subspecies was comparable to that observed among other well-established parrot species. For example, the average pairwise genetic distance for *P*. *r*. *robustus* vs. *P*. *r*. *fuscicollis* (D = 4.5%) and *P*. *r*. *robustus* vs. *P*. *r*. *suahelicus* (D = 4.9%; Table 3) was greater than the genetic difference between three well-established cockatoo species [69]: *Calyptorhynchus funereus* vs. *C*. *latirostris* (D = 3.0%) and *C*. *funereus* vs. *C*. *baudinii* (D = 3.6%; S6 Table). Comparable genetic distance values were observed by Rocha et al. [70] using COI sequences to investigate the taxonomic relationship between two closely related Amazon parrot species, *Amazona pretrei* and *A*. *tucumana* (D = 2.2%).

**Table 3:** The average pairwise genetic distances estimated in RAxML using the concatenated dataset of all three gene regions (below diagonal) and using COI data only (above diagonal) from the *Poicephalus* specimens from the current study.

	*P*. *r*. *robustus*	*P*. *r*. *suahelicus*	*P*. *r*. *fuscicollis*	*P*.*g*. *gulielmi*	*P*.*g*. *massaicus*	*P*. *meyeri*	*P*. *rueppellii*	*P*. *cryptoxanthus*
*P*. *robustus robustus*	*	0.049	0.045	0.237	0.277	0.26	0.281	0.238
*P*. *r*. *suahelicus*	0.036	*	0.009	0.27	0.313	0.242	0.239	0.211
*P*. *r*. *fuscicollis*	0.045	0.009	*	0.261	0.289	0.262	0.258	0.217
*P*. *gulielmi gulielmi*	0.175	0.185	0.189	*	0.074	0.406	0.285	0.316
*P*. *g*. *massaicus*	0.164	0.164	0.186	0.044	*	0.485	0.376	0.432
*P*. *meyeri*	0.271	0.243	0.247	0.35	0.392	*	0.113	0.124
*P*. *rueppellii*	0.288	0.243	0.248	0.271	0.325	0.104	*	0.115
*P*. *cryptoxanthus*	0.241	0.208	0.205	0.263	0.325	0.11	0.105	*

#### Molecular clock analysis

The molecular clock analyses conducted with the concatenated (COI, 16S rRNA and β-fib) and mtDNA (COI and 16S rRNA) datasets produced similar maximum clade probability trees, which suggests that the β-fib missing data did not negatively bias the molecular clock analysis. The estimated divergence dates obtained from the Schweizer et al. [61] and White et al. [62] calibration points were similar (all fall within the 95% highest posterior density (HPD) range of each other; Table 2). The most recent common ancestor of the *Poicephalus* species included in the present study is 10.27 to 10.63 Mya. The origin of the *P*. *robustus* clade is estimated at 6.16 to 6.72 Mya. The maximum clade probability tree suggests that the *P*. *r*. *fuscicollis* and *P*. *r*. *suahelicus* lineage (0.57 to 0.69 Mya) is younger than the most recent common ancestor of the three *P*. *r*. *robustus* populations (Eastern Cape, KwaZulu-Natal and Limpopo; 1.16 to 1.44 Mya). It is clear that *P*. *r*. *robustus* represents a distinct evolutionary lineage. Having diverged from the other species during the late Pliocene to early Pleistocene (2.13 to 2.67 Mya; Table 2). Similar divergence dates were estimated for two recognised cockatoo species, *Calyptorhynchus funereus* and *Calyptorhynchus latirostris* (2.49 to 2.83 Mya; S1 Fig).

## Discussion

The multilocus nuclear and mtDNA results obtained from the current study along with previous morphological, ecological and behavioural data [10–13] provide strong support for the classification of *P*. *r*. *robustus* as a distinct species separate from *P*. *r*. *fuscicollis* and *P*. *r*. *suahelicus*, namely *P*. *robustus* sensu stricto. Our results showed no hybrids or signs of genetic introgression among *P*. *r*. *robustus* and *P*. *r*. *suahelicus*, even in the Limpopo Province of South Africa where these subspecies occur in close proximity.

Multilocus molecular data is often used to investigate taxonomic issues within Psittaciformes. Wenner et al. [22] performed a taxonomic analysis of the *Amazona farinose* species complex using four mtDNA and two non-coding nuclear intron fragments. The authors found support for distinct Central and South American Mealy Amazon clades. It was suggested that these clades should be split into separate species to allow for the implementation of appropriate conservation planning [22]. In another study, the phylogenetic relationships within the *Ampazona ochrocephala* species complex were investigated by Eberhard et al. [32] using four mtDNA markers. The authors found no support for the division of the complex into three species as proposed by others [32, 71–73]. Molecular data have also been used in the past to resolve taxonomic problems in other avian species. For example, microsatellite and mtDNA data were used to assess taxonomic questions within the widespread plover species, *Charadrius alexandrinus* [74]. The authors confirmed the recommendations by Küpper et al. [75] that *C*. *nivosus* should be considered a separate species. Vilaça and Santos [76] investigated the taxonomic status of the *Basileuterus culicivorus* species complex using an mtDNA (cytochrome *b*), a nuclear intron (β-fibrinogen intron 5) and six microsatellite markers. The two species from the species complex were found to be genetically indistinguishable and it was recommended that these taxa should be grouped into a single species, namely *Basileuterus culicivorus*.

### Evolution of the Cape Parrot

The divergence of *P*. *robustus* senso stricto from *P*. *r*. *fuscicollis* and *P*. *r*. *suahelicus* coincides with the start of the Quaternary Period (early Pleistocene) about 2.4 Mya. The Quaternary period consisted of a series of ice ages which led to major global climatic changes resulting in drastic vegetation and habitat changes. These climatic changes led to numerous cycles of grassland expansions and forest contractions and *vice versa*. The expansion of grasslands in Africa and subsequent forest contraction happened at around 2.4 Mya [77]. It has been estimated that the Afromontane forests of southern Africa have been expanding and contracting over the last 100 000 years in accordance with glacial cycles [78]. South African Afromontane forest is the oldest of the two major forest types found in southern Africa, and has been present prior to the last glacial maximum (~18000 BP) [79]. The discovery of parrot fossils in the Western Cape Province, South Africa, dating to the early Pliocene indicates the presence of woodlands in this area and signifies the substantial changes in habitat during the Plio-Pleistocene [80]. The contraction of forests during the arid glacial periods would have driven ancestral forest dwelling species, for example the *P*. *robustus* ancestor, into forest refugia [81]. These fragmented subpopulations would have started to differentiate under adaptive pressures (such as dietary constraints), leading to speciation events [9, 11]. It is proposed that about 1–2 million years (Myr) is sufficient time for speciation to occur [81]. Using *Cytochrome-b* sequence data, Kundu et al. [82] estimated that speciation events occurred within the Afro-Asian parakeet genus *Psittacula* about every 1–2 Myr, and our data suggests that *Poicephalus* show similar short periods of cladogenesis. Comparable molecular clock estimates were obtained for the two extant New Zealand *Nestor* species (*Nestor meridionalis* and *Nestor notabili*; [83]). The authors suggest that the separation between *N*. *meridionalis* and *N*. *notabili* occurred between 2.3 to 2.5 Mya, using a multilocus dataset and calibration points from Schweizer et al. [61] and White et al. [62].

### Taxonomic and conservation considerations

Multiple data sources, including morphological, ecological, behavioural and now molecular, provide convincing scientific evidence that *P*. *r*. *robustus* is a distinct taxonomic unit separate from *P*. *r*. *fuscicollis* and *P*. *r*. *suahelicus*. This lineage fulfills the criteria for at least four methods of species delimitation including Biological [84], Morphological [85, 86], Genotypic [87] and Phylogenetic [88, 89] species concepts. Reproductive isolation is a key criteria for the Biological Species Concept. Behavioural studies have reported that the two taxa whose distributions overlap in South Africa, *P*. *r*. *robustus* and *P*. *r*. *suahelicus*, breed at different times of the year, with *P*. *r*. *robustus* breeding from August to February (mainly utilizing *Afrocacarpus/Podocarpus* trees), while *P*. *r*. *suahelicus* breeds from April to August (preferring *Adansonia* trees for nesting) [20, 21]. In addition, the genetic data presented in this study found no signs of introgression between these two taxa, providing additional evidence for reproductive isolation. Morphologically, *P*. *r*. *robustus* can be easily seperated from the other subspecies, with distinctive colouration [10, 13, 14], small body size and much smaller, narrower bill [13]. These unique diagnostic morphological characters support the reclassification of this taxon using the Morphological Species Concept. The elevation of *P*. *r*. *robustus* to species is also supported by the genetic differentiation of this taxon from other *Poicephalus* species using both microsatellite and sequence data. The mutilocus genotype data unambiguously assigned *P*. *r*. *robustus* individuals to a single genetic cluster separate from other *Poicephalus* species and subspecies. This finding is further strengthened by phylogenetic analysis of both mtDNA and nuclear sequences, with *P*. *r*. *robustus* recovered as monophyletic on both the maximum likelihood and Bayesian trees. The observed molecular divergence between *P*. *r*. *robustus* and other subspecies is congruent with that seperating other well-established parrot species. The *P*. *r*. *robustus* lineage can also be diagnosed by fixed molecular characters (three synapomophic and eleven autapomorphic). Molecular clock analysis suggests that the *P*. *r*. *robustus* lineage diverged from *P*. *r*. *fuscicollis* and *P*. *r*. *suahelicus* approximately 2.4 Mya (early Pleistocene).

We propose that *P*. *r*. *robustus* should be elevated to species status, namely *P*. *robustus* sensu stricto following Gmelin [90]. Given that our molecular data support a close relationship between *P*. *r*. *fuscicollis* and *P*. *r*. *suahelicus*, we recommend that these two taxa remain as subspecies under *P*. *fuscicollis*, namely *P*. *f*. *fuscicollis* stat. nova. [91] and *P*. *f*. *suahelicus* stat. nova. [92] following previous authors [10, 11, 13]. The reclassification of *P*. *robustus* will have considerable implications for conservation management. Given that the South African Cape Parrot (*P*. *robustus* sensu stricto) has a population size less than 10 000 mature individuals with no subpopulation containing more than 1000 mature individuals [23, 93] *P*. *robustus* sensu stricto meets criterion C2a(i) for a Vulnerable listing in the IUCN Red List of Threatened Species. The Cape Parrot also meets all of the biological criteria for a CITES Appendix I listing [11]. The recognition of *P*. *robustus* sensu stricto will allow for the effective regulation and monitoring of any international trade under CITES.

## Conclusion

Our study is the most comprehensive analysis of the taxonomic relationships within the *P*. *robustus* clade using molecular data. The clear genetic differentiation of *P*. *r*. *robustus* from *P*. *r*. *fuscicollis* and *P*. *r*. *suahelicus* coupled with the differences in morphology, habitat and dietary needs provides strong scientific evidence for the elevation of *P*. *r*. *robustus* to *P*. *robustus* sensu stricto. Our results are sufficient to provide conservation authorities with strong evidence that the South African endemic Cape Parrot should be viewed as a Vulnerable species of conservation priority. This recognition will in turn assist the biodiversity conservation sector to prioritize, plan and implement conservation strategies.

## Supporting Information

S1 Fig The maximum clade probability trees generated using the concatenated and mtDNA only data sets, with two sets of calibration points each. (DOCX)

S1 Table The *Poicephalus* specimens included in the present study. (DOCX)

S2 Table Genbank accession numbers for *Poicephalus* sequences generated in the present study and accession numbers for the 27 additional parrot sequences used for molecular clock analysis. (DOCX)

S3 Table The polymorphic information content and null allele frequencies of each locus over all *Poicephalus* specimens analysed. (XLSX)

S4 Table The pairwise F_ST_ values for all *Poicephalus* specimens used in this study. (DOCX)

S5 Table Summary statistics of the COI, 16S rRNA and β-fib sequences generated for the *Poicephalus* specimens analysed in the current study. (DOCX)

S6 Table The COI pairwise genetic distances calculated in RAxML for four *Calyptorhynchus* sp. sequences. (DOCX)
