# Supplementary material for: Molecular Systematics of the Cape Parrot (Poicephalus robustus): Implications for Taxonomy and Conservation
Source: PLoS One. 2015 Aug 12;10(8):e0133376. doi: 10.1371/journal.pone.0133376 (PMC4534405; doi:10.1371/journal.pone.0133376)
Supplement: S1 Fig — (DOCX) [file pone.0133376.s001.docx]

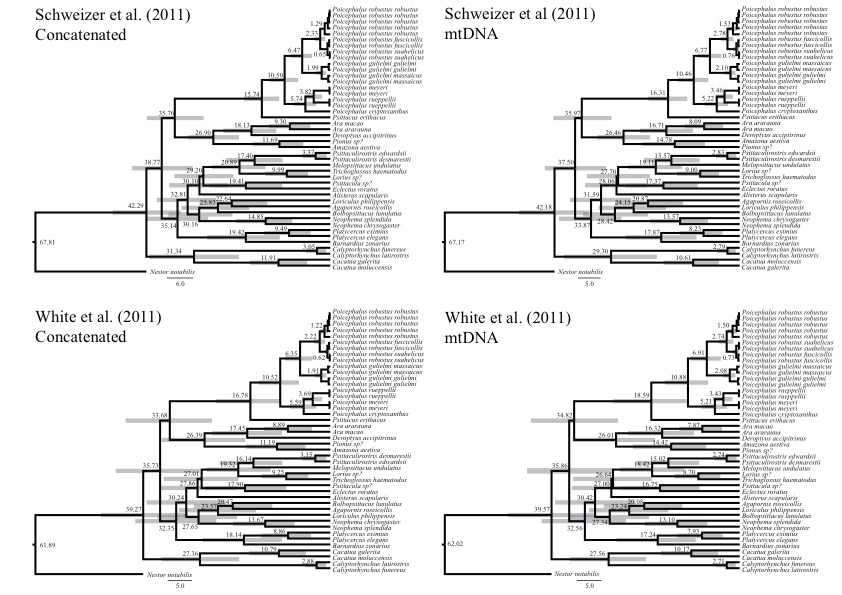


**S1 Fig. Maximum clade probability trees generated using the concatenated data (16S rRNA, COI and β-fib) and mitochondrial DNA (16S rRNA and COI) data. Separate analyses were conducted using Schweizer et al. 2011 and White et al. 2011 calibration points. Values at nodes indicate the posterior mean divergence dates in millions of years before present. Shaded bars indicate the 95% highest posterior density (HPD) credibility intervals.**
