# Supplementary material for: Molecular Systematics of the Cape Parrot (Poicephalus robustus): Implications for Taxonomy and Conservation
Source: PLoS One. 2015 Aug 12;10(8):e0133376. doi: 10.1371/journal.pone.0133376 (PMC4534405; doi:10.1371/journal.pone.0133376)
Supplement: S1 Table — (DOCX) [file pone.0133376.s002.docx]

**S1 Table. The *Poicephalus* specimens included in the present study.** Collection numbers and locations were those assigned to specimens by museums or the collections of researchers. GPS coordinates are taken from the general area of sample collection. The institutions where samples can be accessed are listed. Specimens used for DNA sequencing are indicated by *.

| **Species/Subspecies:** | **Analysis code:** | **Collection numbers:** | **Sampling location:** | **GPS^$^:** | **Sample storage:** | **Sample type:** |
| --- | --- | --- | --- | --- | --- | --- |
| *P. robustus robustus* | Prob01 | FH50* | Alice, Eastern Cape Province, RSA | -32.796097, 26.850024 | University of KwaZulu-Natal (UKZN) | Whole blood |
|  | Prob02 | LG02 | King Williams Town, Eastern Cape Province, RSA | -32.880202, 27.398856 | UKZN | Whole blood |
|  | Prob03 | KMB638 | Eastern Cape Province, RSA | Unknown | East London Museum (ELM), South Africa | Archival museum sample |
|  | Prob04 | 7201 | Cambridge district, East London, Eastern Cape Province, RSA | -33.008834, 27.802254 | ELM | Archival museum sample |
|  | Prob05 | 13276 | Lusikisiki, Wild Coast, Eastern Cape Province, RSA | -31.366218, 29.570018 | ELM | Archival museum sample |
|  | Prob06 | 13277 | Lusikisiki, Wild Coast, Eastern Cape Province, RSA | -31.366218, 29.570018 | ELM | Archival museum sample |
|  | Prob07 | 16100 | Frankfort, Eastern Cape Province, RSA | -32.720507, 27.453272 | ELM | Archival museum sample |
|  | Prob08 | 16104 | King William’s Town, Eastern Cape Province, RSA | -32.880202, 27.398856 | ELM | Archival museum sample |
|  | Prob09 | 16105 | Pirie hatchery, Eastern Cape Province, RSA | -32.791100, 27.247902 | ELM | Archival museum sample |
|  | Prob10 | 16106 | King William’s Town, Eastern Cape Province, RSA | -32.880202, 27.398856 | ELM | Archival museum sample |
|  | Prob11 | KZNT01* | Creighton, KwaZulu-Natal, RSA | -30.027832, 29.838148 | UKZN | Muscle tissue |
|  | Prob12 | KZNT02* | Creighton, KwaZulu-Natal, RSA | -30.027832, 29.838148 | UKZN | Muscle tissue |
|  | Prob13 | KZNT03 | Creighton, KwaZulu-Natal, RSA | -30.027832, 29.838148 | UKZN | Muscle tissue |
|  | Prob14 | KZNT04 | Creighton, KwaZulu-Natal, RSA | -30.027832, 29.838148 | UKZN | Muscle tissue |
|  | Prob15 | KZNT05 | Creighton, KwaZulu-Natal, RSA | -30.027832, 29.838148 | UKZN | Muscle tissue |
|  | Prob16 | KZNT06 | Creighton, KwaZulu-Natal, RSA | -30.027832, 29.838148 | UKZN | Muscle tissue |
|  | Prob17 | CR01 | Creighton, KwaZulu-Natal, RSA | -30.027832, 29.838148 | UKZN | Whole blood |
|  | Prob18 | CR02 | Creighton, KwaZulu-Natal, RSA | -30.027832, 29.838148 | UKZN | Whole blood |
|  | Prob19 | CR03 | Creighton, KwaZulu-Natal, RSA | -30.027832, 29.838148 | UKZN | Whole blood |
|  | Prob20 | CR04 | Creighton, KwaZulu-Natal, RSA | -30.027832, 29.838148 | UKZN | Whole blood |
| **S1 Table** (Continued) |  |  |  |  |  |  |
| **Species/Subspecies:** | **Analysis code:** | **Collection numbers:** | **Sampling location:** | **GPS^$^:** | **Sample storage:** | **Sample type:** |
|  | Prob21 | CR05 | Creighton, KwaZulu-Natal, RSA | -30.027832, 29.838148 | UKZN | Feather |
| *P. robustus robustus* | Prob22 | CR06 | Creighton, KwaZulu-Natal, RSA | -30.027832, 29.838148 | UKZN | Feather |
|  | Prob23 | CR07 | Creighton, KwaZulu-Natal, RSA | -30.027832, 29.838148 | UKZN | Feather |
|  | Prob24 | RMO1* | Tzaneen, Limpopo, RSA | -23.859859, 30.006596 | UKZN | Whole blood |
|  | Prob25 | RMO2* | Tzaneen, Limpopo, RSA | -23.859859, 30.006596 | UKZN | Whole blood |
|  | Prob26 | Pool1 | Tzaneen, Limpopo, RSA | -23.859859, 30.006596 | UKZN | Feather |
|  | Prob27 | Pool2 | Tzaneen, Limpopo, RSA | -23.859859, 30.006596 | UKZN | Feather |
|  | Prob28 | TMIIa 2078 | Limpopo, RSA | -23.822019, 30.131136 | Ditsong National Museum of Natural History (DNM), South Africa | Archival museum sample |
|  | Prob29 | TM 16406 | Limpopo, RSA | -23.822019, 30.131136 | DNM | Archival museum sample |
|  | Prob30 | TM 16407 | Limpopo, RSA | -23.822019, 30.131136 | DNM | Archival museum sample |
|  | Prob31 | TM 25266 | Limpopo, RSA | -23.822019, 30.131136 | DNM | Archival museum sample |
|  | Prob32 | TM80817 | Magoebaskloof, Limpopo, RSA | -23.822019, 30.131136 | DNM | Archival museum sample |
| *P. r. suahelicus* | Prs01 | Prs 1 | Unknown | Unknown | UKZN | Whole blood |
|  | Prs02 | Prs 2 | Unknown | Unknown | UKZN | Whole blood |
|  | Prs03 | Prs 3 | Unknown | Unknown | UKZN | Whole blood |
|  | Prs04 | Prs 4 | Unknown | Unknown | UKZN | Whole blood |
|  | Prs05 | Prs 24 | Unknown | Unknown | UKZN | Whole blood |
|  | Prs06 | P.f.suah1 | Unknown | Unknown | UKZN | Whole blood |
|  | Prs07 | P.f.suah2 | Unknown | Unknown | UKZN | Whole blood |
|  | Prs08 | P.f.suah3 | Unknown | Unknown | UKZN | Whole blood |
|  | Prs09 | P.f.suah4 | Unknown | Unknown | UKZN | Whole blood |
|  | Prs10 | P.f.suah5 | Unknown | Unknown | UKZN | Whole blood |
|  | Prs11 | P.f.suah6 | Unknown | Unknown | UKZN | Whole blood |
|  | Prs12 | 20814 | Captive bred | CB | Loro Parque Foundation (LPF), Spain | Whole blood |
|  | Prs13 | 20815 | Captive bred | CB | LPF | Whole blood |
| **S1 Table** (Continued) |  |  |  |  |  |  |
| **Species/Subspecies:** | **Analysis code:** | **Collection numbers:** | **Sampling location:** | **GPS^$^:** | **Sample storage:** | **Sample type:** |
| *P. r. suahelicus* | Prs14 | 8269* | Zambia | Unknown | National Zoological Gardens (NZG), South Africa | Whole blood |
|  | Prs15 | 8270* | Zambia | Unknown | NZG | Whole blood |
|  | Prs16 | 8271 | Zambia | Unknown | NZG | Whole blood |
|  | Prs17 | 8272 | Zambia | Unknown | NZG | Whole blood |
|  | Prs18 | 8273 | Zambia | Unknown | NZG | Whole blood |
|  | Prs19 | 8274 | Zambia | Unknown | NZG | Whole blood |
|  | Prs20 | 8275 | Zambia | Unknown | NZG | Whole blood |
|  | Prs21 | CS01 | Levubu, Limpopo, RSA | -23.109336, 30.323897 | NZG | Feather |
|  | Prs22 | CS03 | Levubu, Limpopo, RSA | -23.109336, 30.323897 | NZG | Feather |
|  | Prs23 | CS04 | Levubu, Limpopo, RSA | -23.109336, 30.323897 | NZG | Feather |
| *P. r. fuscicollis* | Prf01 | 249 ♂ | Unknown | Unknown | NZG | Whole blood |
|  | Prf02 | 249 ♀ | Unknown | Unknown | NZG | Whole blood |
|  | Prf03 | 383 ♂ | Unknown | Unknown | NZG | Whole blood |
|  | Prf04 | 383 ♀* | Unknown | Unknown | NZG | Whole blood |
|  | Prf05 | 386 ♂* | Unknown | Unknown | NZG | Whole blood |
|  | Prf06 | 386 ♀ | Unknown | Unknown | NZG | Whole blood |
|  | Prf07 | 388 ♂ | Unknown | Unknown | NZG | Whole blood |
|  | Prf08 | 388 ♀ | Unknown | Unknown | NZG | Whole blood |
|  | Prf09 | 1423 ♂ | Unknown | Unknown | NZG | Whole blood |
|  | Prf10 | 1423 ♀ | Unknown | Unknown | NZG | Whole blood |
|  | Prf11 | 1440 | Captive bred | CB | LPF | Whole blood |
|  | Prf12 | 1441 | Captive bred | CB | LPF | Whole blood |
|  | Prf13 | 1514 | Captive bred | CB | LPF | Whole blood |
|  | Prf14 | 8639 | Captive bred | CB | LPF | Whole blood |
|  | Prf15 | 11065 | Captive bred | CB | LPF | Whole blood |
|  | Prf16 | 11068 | Captive bred | CB | LPF | Whole blood |
|  |  |  |  |  |  |  |
| **S1 Table** (Continued) |  |  |  |  |  |  |
| **Species/Subspecies:** | **Analysis code:** | **Collection numbers:** | **Sampling location:** | **GPS^$^:** | **Sample storage:** | **Sample type:** |
| *P. r. fuscicollis* | Prf17 | 17305 | Captive bred | CB | LPF | Whole blood |
|  | Prf18 | 24091 | Captive bred | CB | LPF | Whole blood |
|  | Prf19 | 25200 | Captive bred | CB | LPF | Whole blood |
|  | Prf20 | 25932 | Captive bred | CB | LPF | Whole blood |
|  | Prf21 | 25960 | Captive bred | CB | LPF | Whole blood |
|  | Prf22 | BMNH 1929.2.18.110 | Farafenni, N.B.Prov., Gambia | 13.566788, -15.599320 | British Museum of Natural History at Tring (BMNH), United Kingdom | Archival museum sample |
|  | Prf23 | BMNH 1929.2.18.112 | Kerewan, N.B.Prov., Gambia | 13.490150, -16.085272 | BMNH | Archival museum sample |
|  | Prf24 | BMNH 1929.2.18.114 | Farafenni, N.B.Prov., Gambia | 13.566788, -15.599320 | BMNH | Archival museum sample |
|  | Prf25 | BMNH 1929.2.18.109 | Kerewan, N.B.Prov., Gambia | 13.490150, -16.085272 | BMNH | Archival museum sample |
|  | Prf26 | BMNH 1910.5.6.147 | Guinea-Bissau | Unknown | BMNH | Archival museum sample |
| *P. rueppellii* | Prup01 | 8250 | Swakop River, Namibia | -22.651557, 14.624207 | Durban Natural Sciences Museum (DNSM), South Africa | Archival museum sample |
|  | Prup02 | 8251 | Swakop River, Namibia | -22.651557, 14.624207 | DNSM | Archival museum sample |
|  | Prup03 | 8252 | Swakop River, Namibia | -22.651557, 14.624207 | DNSM | Archival museum sample |
|  | Prup04 | 8253 | Swakop River, Namibia | -22.651557, 14.624207 | DNSM | Archival museum sample |
|  | Prup05 | 8254 | Swakop River, Namibia | -22.651557, 14.624207 | DNSM | Archival museum sample |
|  | Prup06 | 8255 | Swakop River, Namibia | -22.651557, 14.624207 | DNSM | Archival museum sample |
|  | Prup07 | 8256 | Swakop River, Namibia | -22.651557, 14.624207 | DNSM | Archival museum sample |
|  | Prup08 | 8257 | Okahandja, Namibia | -21.983572, 16.916715 | DNSM | Archival museum sample |
|  | Prup09 | 8258 | Windhoek, Namibia | -22.566294, 17.059741 | DNSM | Archival museum sample |
|  | Prup10 | 8259 | Klipkop Farm, South of Otjiwarongo, Namibia | -20.667061, 16.742880 | DNSM | Archival museum sample |
|  |  |  |  |  |  |  |
| **S1 Table** (Continued) |  |  |  |  |  |  |
| **Species/Subspecies:** | **Analysis code:** | **Collection numbers:** | **Sampling location:** | **GPS^$^:** | **Sample storage:** | **Sample type:** |
| *P. rueppellii* | Prup11 | 8260 | Klipkop Farm, South of Otjiwarongo, Namibia | -20.667061, 16.742880 | DNSM | Archival museum sample |
|  | Prup12 | 8261 | Okahandja, Namibia | -21.983572, 16.916715 | DNSM | Archival museum sample |
|  | Prup13 | Pru01* | Captive bred | CB | UKZN | Whole blood |
|  | Prup14 | Pru02* | Captive bred | CB | UKZN | Whole blood |
|  | Prup15 | Pru03 | Captive bred | CB | UKZN | Whole blood |
|  | Prup16 | Pru04 | Captive bred | CB | UKZN | Whole blood |
| *P. meyeri* | Pm01 | Pm01* | Captive bred | CB | UKZN | Whole blood |
|  | Pm02 | Pm02* | Captive bred | CB | UKZN | Whole blood |
|  | Pm03 | Pm03 | Captive bred | CB | UKZN | Whole blood |
|  | Pm04 | Pm04 | Captive bred | CB | UKZN | Whole blood |
|  | Pm05 | 16406 | Sentinel Ranch, Beit Bridge, Zimbabwe | -22.2167, 30 | DNSM | Archival museum sample |
|  | Pm06 | 29461 | Humani Ranch, Sabi Valley, Zimbabwe | -20.491367, 32.242027 | DNSM | Archival museum sample |
|  | Pm07 | 32040 | Okavango River, Andara, Namibia | -18.061680, 21.443950 | DNSM | Archival museum sample |
|  | Pm08 | 32041 | Okavango River, Andara, Namibia | -18.061680, 21.443950 | DNSM | Archival museum sample |
|  | Pm09 | 8272 | Victoria Falls, Zimbabwe | -17.907320, 25.821980 | DNSM | Archival museum sample |
|  | Pm10 | 25474 | Northam, South Africa | -24.950103, 27.267488 | DNSM | Archival museum sample |
|  | Pm11 | 29462 | Humani Ranch, Sabi Valley, Zimbabwe | -20.491367, 32.242027 | DNSM | Archival museum sample |
|  | Pm12 | 29463 | Humani Ranch, Sabi Valley, Zimbabwe | -20.491367, 32.242027 | DNSM | Archival museum sample |
| *P. cryptoxanthus* | Pcryp01 | 11920 | N of Siteki, Swaziland | -26.449321, 31.949929 | DNSM | Archival museum sample |
|  | Pcryp02 | 20733 | Chimonzo, Mozambique | -24.9483, 33.2917 | DNSM | Archival museum sample |
|  | Pcryp03 | 20734 | Chimonzo, Mozambique | -24.9483, 33.2917 | DNSM | Archival museum sample |
|  | Pcryp04 | 20735 | Massinga, Mozambique | -23.332600, 35.385160 | DNSM | Archival museum sample |
| **S1 Table** (Continued) |  |  |  |  |  |  |
| **Species/Subspecies:** | **Analysis code:** | **Collection numbers:** | **Sampling location:** | **GPS^$^:** | **Sample storage:** | **Sample type:** |
| *P. cryptoxanthus* | Pcryp05 | 23857 | Inhaminga, Mozambique | -18.416012, 35.024962 | DNSM | Archival museum sample |
|  | Pcryp06 | 26840 | Massinga, Mozambique | -23.332600, 35.385160 | DNSM | Archival museum sample |
|  | Pcryp07 | 26841 | Massinga, Mozambique | -23.332600, 35.385160 | DNSM | Archival museum sample |
|  | Pcryp08 | 8238 | Newington, Malamala, South Africa | -24.805498, 31.540776 | DNSM | Archival museum sample |
|  | Pcryp09 | 8239 | Newington, Malamala, South Africa | -24.805498, 31.540776 | DNSM | Archival museum sample |
|  | Pcryp10 | 8240 | Newington, Malamala, South Africa | -24.805498, 31.540776 | DNSM | Archival museum sample |
|  | Pcryp11 | 8241 | Newington, Malamala, South Africa | -24.805498, 31.540776 | DNSM | Archival museum sample |
|  | Pcryp12 | 8242 | Newington, Malamala, South Africa | -24.805498, 31.540776 | DNSM | Archival museum sample |
|  | Pcryp13 | 8243 | Newington, Malamala, South Africa | -24.805498, 31.540776 | DNSM | Archival museum sample |
|  | Pcryp14 | 8248 | Chimonzo, Mozambique | -24.9483, 33.2917 | DNSM | Archival museum sample |
|  |  | URB Pcryp1* | Captive bred | CB | UKZN | Whole blood |
| *P.g.massaicus* | Pgm01 | 8263 | Naro Moru, Mt. Kenya, Kenya | -0.159133, 37.313424 | DNSM | Archival museum sample |
|  | Pgm02 | 8264 | Naro Moru, Mt. Kenya, Kenya | -0.159133, 37.313424 | DNSM | Archival museum sample |
|  | Pgm03 | 8265 | Molo, Kenya | -0.249101, 35.732303 | DNSM | Archival museum sample |
|  | Pgm04 | 38005 | Captive bred | CB | DNSM | Archival museum sample |
|  | Pgm05 | 38006 | Captive bred | CB | DNSM | Archival museum sample |
|  | Pgm06 | Pgm01* | Captive bred | CB | UKZN | Whole blood |
|  | Pgm07 | Pgm02* | Captive bred | CB | UKZN | Whole blood |
|  | Pgm08 | Pgm03 | Captive bred | CB | UKZN | Whole blood |
|  | Pgm09 | Pgm04 | Captive bred | CB | UKZN | Whole blood |
|  | Pgm10 | Pgm05 | Captive bred | CB | UKZN | Whole blood |
|  |  |  |  |  |  |  |
| **S1 Table** (Continued) |  |  |  |  |  |  |
| **Species/Subspecies:** | **Analysis code:** | **Collection numbers:** | **Sampling location:** | **GPS^$^:** | **Sample storage:** | **Sample type:** |
| *P.g.massaicus* | Pgm11 | Pgm06 | Captive bred | CB | UKZN | Whole blood |
| *P.g.gulielmi* | Pgg01 | Pgg01* | Captive bred | CB | UKZN | Whole blood |
|  | Pgg02 | Pgg02* | Captive bred | CB | UKZN | Whole blood |
|  | Pgg03 | Pgg03 | Captive bred | CB | UKZN | Whole blood |
|  | Pgg04 | Pgg04 | Captive bred | CB | UKZN | Whole blood |

*Samples used for sequencing of COI, 16S rRNA and β-fibrinogen gene regions

^$^ GPS coordinates for museum samples were estimated using the provided locality information

CB = Captive bred
