# Supplementary material for: Molecular Systematics of the Cape Parrot (Poicephalus robustus): Implications for Taxonomy and Conservation
Source: PLoS One. 2015 Aug 12;10(8):e0133376. doi: 10.1371/journal.pone.0133376 (PMC4534405; doi:10.1371/journal.pone.0133376)
Supplement: S2 Table — (DOCX) [file pone.0133376.s003.docx]

**S2 Table. Genbank accession numbers for *Poicephalus* sequences generated in the present study and accession numbers for the 27 additional parrot sequences used for molecular clock analysis.**

| **Species:** | **Accession numbers:** | | |
| --- | --- | --- | --- |
|  | **16S rRNA** | **β-fib** | **COI** |
| *Poicephalus robustus robustus1* | KP856844 | KP856857 | KP856872 |
| *P. r. robustus2* | KP856845 | - | KP856873 |
| *P. r. robustus3* | KP856846 | KP856858 | KP856874 |
| *P. r. robustus4* | KP856847 | KP856859 | KP856875 |
| *P. r. robustus5* | KP856848 | KP856860 | KP856876 |
| *P. r. suahelicus1* | KP856849 | KP856861 | KP856877 |
| *P. r. suahelicus2* | KP856850 | KP856862 | KP856878 |
| *P. r. fuscicollis1* | KP856842 | KP856856 | KP856870 |
| *P. r. fuscicollis2* | KP856843 | - | KP856871 |
| *P. rueppellii1* | KP856851 | - | KP856879 |
| *P. rueppellii2* | KP856852 | - | KP856880 |
| *P. meyeri1* | KP856840 | - | KP856868 |
| *P. meyeri2* | KP856841 | - | KP856869 |
| *P. cryptoxanthus* | KP856835 | - | KP856863 |
| *P. gulielmi gulielmi1* | KP856836 | - | KP856864 |
| *P. gulielmi2* | KP856837 | KP856853 | KP856865 |
| *P.g.massaicus1* | KP856838 | KP856854 | KP856866 |
| *P.g.massaicus2* | KP856839 | KP856855 | KP856867 |
| *Agapornis roseicollis* | EU410486.1 | GQ395348.1 | EU410486.1 |
| *Alisterus scapularis* | EU197096.1 | EU739363.1 | JN801395.1 |
| *Amazona* *aestiva* | EU197111.1 | AY301472.1 | FJ027055.1 |
| *Ara* *ararauna* | - | AY301514.1 | FJ808626.1 |
| *Ara* *macao* | EF635432.1 | - | JN801493.1 |
| *Barnardius* *zonarius* | - | - | JN801399.1 |
| *Bolbopsittacus* *lunulatus* | - | - | KC354896.1 |
| *Cacatua* *galerita* | - | - | JN801403.1 |
| *Cacatua* *moluccensis* | JF414239.1 | - | JF414239.1 |
| *Calyptorhynchus* *funereus* | - | AY695167.1 | JF414279.1 |
| *Calyptorhynchus* *latirostris* | EU197114.1 | - | JF414274.1 |
| *Deroptyus* *accipitrinus* | - | - | JQ174682.1 |
| *Eclectus* *roratus* | EU197113.1 | - | JN801439.1 |
| *Loriculus* *philippensis* | - | - | KC354935.1 |
| *Lorius* *sp*. | EU197097.1  (*Lorius lory*) | - | JQ175284.1  (*Lorius albidinucha*) |
| *Melopsittacus* *undulatus* | EF450826.1 | - | F450826.1 |
| *Neophema* *chrysogaster* | JX133087.1 | - | JX133087.1 |
| *Neophema* *splendida* | EU197100.1 | - | JQ175546.1 |
| *Nestor* *notabilis* | EU197116.1 | - | HQ616639.1 |
| *Pionus* *sp*. | EU197112.1  (*Pionus* *menstruus*) | AY301516.1  (*Pionus* *menstruus*) | JQ175856.1  (*Pionus* *fuscus*) |
| *Platycercus* *elegans* | - | EU739470.1 | JQ175887.1 |
| *Platycercus* *eximius* | EU197095.1 | - | JQ175889.1 |
| *Psittacula* *sp*. | EU197107.1  (*Psittacula* *cyanocephala*) | EU739474.1  (*Psittacula* *alexandri*) | KC439335.1  (*Psittacula* *eupatria*) |
| *Psittaculirostris* *desmarestii* | EU197121.1 | - | - |
| *Psittaculirostris* *edwardsii* | EU197117.1 | - | - |
| *Psittacus* *erithacus* | EU197109.1 | AY301518.1 | KF381364.1 |
| *Trichoglossus* *haematodus* | - | - | JN801465.1 |
