# Supplementary material for: Molecular Systematics of the Cape Parrot (Poicephalus robustus): Implications for Taxonomy and Conservation
Source: PLoS One. 2015 Aug 12;10(8):e0133376. doi: 10.1371/journal.pone.0133376 (PMC4534405; doi:10.1371/journal.pone.0133376)
Supplement: S4 Table — (DOCX) [file pone.0133376.s005.docx]

**S4 Table: The pairwise F_ST_ values for all *Poicephalus* specimens used in this study.** The F_ST_ values are below diagonal and probability values (*P*-values) above diagonal.

|  | *P. robustus robustus* | *P. r. suahelicus* | *P. r. fuscicollis* | *P. rueppellii* | *P. meyeri* | *P. cryptoxanthus* | *P. gulielmi massaicus* | *P. g. gulielmi* |
| --- | --- | --- | --- | --- | --- | --- | --- | --- |
| P. robustus robustus | * | 0.001 | 0.001 | 0.001 | 0.001 | 0.001 | 0.001 | 0.001 |
| P. r. suahelicus | 0.14 | * | 0.001 | 0.001 | 0.001 | 0.001 | 0.001 | 0.001 |
| P. r. fuscicollis | 0.22 | 0.16 | * | 0.001 | 0.001 | 0.001 | 0.001 | 0.001 |
| P. rueppellii | 0.28 | 0.23 | 0.31 | * | 0.001 | 0.001 | 0.001 | 0.001 |
| P. meyeri | 0.18 | 0.13 | 0.24 | 0.13 | * | 0.001 | 0.001 | 0.001 |
| P. cryptoxanthus | 0.19 | 0.15 | 0.25 | 0.21 | 0.14 | * | 0.001 | 0.001 |
| P. gulielmi massaicus | 0.37 | 0.33 | 0.4 | 0.41 | 0.32 | 0.36 | * | 0.001 |
| P. g. gulielmi | 0.36 | 0.29 | 0.39 | 0.38 | 0.29 | 0.33 | 0.25 | * |
