# Supplementary material for: Molecular Systematics of the Cape Parrot (Poicephalus robustus): Implications for Taxonomy and Conservation
Source: PLoS One. 2015 Aug 12;10(8):e0133376. doi: 10.1371/journal.pone.0133376 (PMC4534405; doi:10.1371/journal.pone.0133376)
Supplement: S5 Table — (DOCX) [file pone.0133376.s006.docx]

**S5 Table. Summary statistics of the COI, 16S rRNA and β-fib sequences generated for the *Poicephalus* specimens analysed in the current study.**

| **Marker name:** | **Number of sequences:** | **Base pair length:** | **Number of variable sites:** | **Number of transitions:** | **Number of transvertions:** | **Number of Indels:** | **T%:** | **C%:** | **A%:** | **G%:** |
| --- | --- | --- | --- | --- | --- | --- | --- | --- | --- | --- |
| **COI** | 18 | 592 | 66 | 54 | 14 | 1 | 24.6 | 35.2 | 24.1 | 16.2 |
| **16S rRNA** | 18 | 535 | 32 | 24 | 8 | 2 | 19.1 | 28.1 | 32.6 | 20.2 |
| **β-fibrinogen** | 10 | 707 | 4 | 3 | 1 | 0 | 33.1 | 22.3 | 26.8 | 17.8 |
