# Supplementary material for: Molecular Systematics of the Cape Parrot (Poicephalus robustus): Implications for Taxonomy and Conservation
Source: PLoS One. 2015 Aug 12;10(8):e0133376. doi: 10.1371/journal.pone.0133376 (PMC4534405; doi:10.1371/journal.pone.0133376)
Supplement: S6 Table — (DOCX) [file pone.0133376.s007.docx]

**S6 Table. The COI pairwise genetic distances calculated in RaxML for four *Calyptorhynchus* sp. sequences.** (Downloaded from GenBank: JF414241.1, JN801424.1, JF414242.1, JF414279.1, JF414274.1).

|  | *C. lathami* | *C. banksii* | *C. baudinii* | *C. funereus* | *C. latirostris* |
| --- | --- | --- | --- | --- | --- |
| *C. lathami* | * |  |  |  |  |
| *C. banksii* | 0.151 | * |  |  |  |
| *C. baudinii* | 0.632 | 0.499 | * |  |  |
| *C. funereus* | 0.686 | 0.627 | 0.036 | * |  |
| *C. latirostris* | 0.594 | 0.497 | 0.009 | 0.03 | * |
